# 

**Published:** 1902-10-25

**Authors:** 


**Notices of Births, Deaths, and Marriages** are inserted in this column at a charge of 2s. 6d. for an announcement not exceeding four lines.

### DEATH.

We regret to announce the death at Forest Hill on the 13th October, of Ellen Mary (née Phillips), wife of Frederick Tingley, after a long illness. Nurse Phillips was late of St. Bartholomew's and University College Hospitals, and up to the time of her marriage took much interest in nursing work at the East End of London. (1015)

### NOTICE TO CORRESPONDENTS.

All MSS., Letters, Books for Review, and other matters intended for the Editor should be addressed The Editor, "The Hospital," The Hospital Building, 28 & 29 Southampton Street, Strand, W.C.
The Editor cannot undertake to return rejected MS., even when accompanied by stamped directed envelope.

**Notice to Advertisers and Subscribers.**

All Advertisements, Orders for Copies of The Hospital, and Business Communications should be addressed to **THE MANAGER, (Not to the Editor)**, The Hospital, 28 & 29 Southampton Street, Strand, London, W.C.
The Cost of Subscription, post free, is as follows.—Per week, 2½d.; per quarter, 2s. 8d.; per half-year, 5s. 3d.; per annum, 10s. 6d. Foreign:—Per annum, 15s. 2d., payable, in all cases, in advance.

**NOTICE.—Vol. XXXII. of "The Hospital" (April to September 1902) in handsome cloth binding will shortly be on sale at the office of this Journal, price, 7s. 6d. Cases for binding the Numbers contained in this Volume 1s. 6d. Index to Vol. XXXII., 2½d., post free.**
**The Monthly Part for November will be ready on November 1. Price 6d., by post 8d.**

### Scraps and Gleanings.

A special feature of the new cottage hospital at St. Andrews is a ward which has been erected as a memorial to the late Lieutenant Tait, the well-known golfer, who was killed at Koodoosberg. A sum of about £1,450 was raised by his admirers, the greater part of which was devoted to endow the ward.

During the three days in one week that the State apartments of Windsor Castle were open to the public about £160 was paid for admission, which will be handed over to local charities.

The cost of the buildings of the new sanatorium at Weymouth, a visit to which was paid recently by the Dowager Duchess of Beaufort, has been about £20,000.

Mr. John Corry, J.P., of Croydon, has given to the Lucknow Soldiers' Home, through the Soldiers and Sailors Homes Fund, a sum of 10,000 rupees as a memorial of his son, Lieut. John B. Corry, R.E., who recently died in India.

### The Student of Medicine.

### APPOINTMENTS.

J. H. Abram, M.D., M.R.C.P.Lond., appointed Honorary Physician to the Liverpool Royal Infirmary. **Donald John Armour**, M.B., M.R.C.P., F.R.C.S., appointed Honorary Surgeon to the St. Marylebone General Dispensary. **J. M. Brydone**, M.R.C.S., L.R.C.P., M.B.Camb., B.C., appointed Resident Medical Officer, Private Wards, Guy's Hospital. **Joseph William Dawes**, M.B., C.M.Edin., appointed Medical Officer of Health for the Borough of Longton. **V. De Boinville**, M.B., B.S.Edin., appointed Assistant House Surgeon to the Salisbury Infirmary. **Joseph P. Frengley**, M.D., B.S., R.U.I., appointed a District Health Officer under the Department of Public Health of the New Zealand Government. **G. D. B. Levick**, L.R.C.P.Lond., M.R.C.S.Eng., appointed District Medical Officer of the Hendon Union. **G. H. Lewis**, M.B., Ch.B.Edin., appointed House Surgeon to the Northampton General Infirmary. **George Okell, jun.**, L.R.C.P.Irel., appointed Certifying Factory Surgeon for the Winsford District of the county of Cheshire. **D. Phillips**, M.R.C.S., L.R.C.P.Lond., appointed Medical Officer to the Great Western Railway Servants at Llandilo. **G. Winfield Roll**, B.A., M.B., B.C.Cantab., F.R.C.S.Eng., appointed Honorary Ophthmalic Surgeon to the St. Mary's Hospital for Sick Children, Plaistow, E. **F. Rowland**, L.R.C.P., L.R.C.S. Edin., appointed District Medical Officer of the Solihull Union. **Harry Shore**, M.D.Durh., D.P.H., appointed Honorary Surgeon to the Walsall and District Hospital. **B. Spearman**, B.C., M.B.Cantab., appointed House Physician to the Northampton General Infirmary. **Geoffrey Stead**, M.D.Brux., L.R.C.P.Lond., M.R.C.S., appointed Honorary Physician to the Walsall and District Hospital. **A. W. H. Walker**, M.D.Brux., L.R.C.P.Lond., M.R.C.S.Eng., appointed Certifying Factory Surgeon for the Harrogate District of the county of York. **Sidney J. Wareham**, F.R.C.S.Eng., L.R.C.P.Lond., L.S.A., appointed Honorary Medical Officer to the St. Mary's Hospital for Sick Children, Plaistow, E.

### "The Hospital" Institutional Library.

"Hospitals and Asylums of the World." 4 Vols., with a Portfolio of Plans, complete £12 12s.

"Burdett's Hospitals and Charities, 1902." 5s.

"Cottage Hospitals, General, Fever, and Convalescent." 10s. 6d.

"Hospital Expenditure: The Commissariat." 2s. 6d.

"A Legal Handbook for the Use of Hospital Authorities.' 2s. 6d.

"The Cottage Hospital Case Book or Register of Patients." 10s and 12s. 6d.

"The Furnishing and Appliances of a Cottage Hospital." 6d.

All these are published by the Scientific Press, Ltd., and may be obtained through any bookseller, or direct from the publishers 28 and 29 Southampton Street, Strand, London, W.C.

